# Activity‐specific metabolic rates for diving, transiting, and resting at sea can be estimated from time–activity budgets in free‐ranging marine mammals

**DOI:** 10.1002/ece3.2546

**Published:** 2017-03-23

**Authors:** Tiphaine Jeanniard‐du‐Dot, Andrew W. Trites, John P. Y. Arnould, John R. Speakman, Christophe Guinet

**Affiliations:** ^1^ Marine Mammal Research UnitInstitute for the Oceans and FisheriesUniversity of British ColumbiaVancouverBCCanada; ^2^ Centre d'Etudes Biologiques de ChizéCNRSVilliers en BoisFrance; ^3^ School of Life and Environmental Sciences (Burwood Campus)Deakin UniversityGeelongAustralia; ^4^ The Institute of Biological and Environmental SciencesAberdeenUK

**Keywords:** Antarctic fur seal, *Arctocephalus gazella*, *Callorhinus ursinus*, diving, energy expenditure, foraging, metabolic rate, northern fur seal, time–activity budget

## Abstract

Time and energy are the two most important currencies in animal bioenergetics. How much time animals spend engaged in different activities with specific energetic costs ultimately defines their likelihood of surviving and successfully reproducing. However, it is extremely difficult to determine the energetic costs of independent activities for free‐ranging animals. In this study, we developed a new method to calculate activity‐specific metabolic rates, and applied it to female fur seals. We attached biologgers (that recorded GPS locations, depth profiles, and triaxial acceleration) to 12 northern (*Callorhinus ursinus*) and 13 Antarctic fur seals (*Arctocephalus gazella*), and used a hierarchical decision tree algorithm to determine time allocation between diving, transiting, resting, and performing slow movements at the surface (grooming, etc.). We concomitantly measured the total energy expenditure using the doubly‐labelled water method. We used a general least‐square model to establish the relationship between time–activity budgets and the total energy spent by each individual during their foraging trip to predict activity‐specific metabolic rates. Results show that both species allocated similar time to diving (~29%), transiting to and from their foraging grounds (~26–30%), and resting (~8–11%). However, Antarctic fur seals spent significantly more time grooming and moving slowly at the surface than northern fur seals (36% vs. 29%). Diving was the most expensive activity (~30 MJ/day if done non‐stop for 24 hr), followed by transiting at the surface (~21 MJ/day). Interestingly, metabolic rates were similar between species while on land or while slowly moving at the surface (~13 MJ/day). Overall, the average field metabolic rate was ~20 MJ/day (for all activities combined). The method we developed to calculate activity‐specific metabolic rates can be applied to terrestrial and marine species to determine the energetic costs of daily activities, as well as to predict the energetic consequences for animals forced to change their time allocations in response to environmental shifts.

## 1 Introduction

Achieving energetic balance is key to survival and successful reproduction for all living beings (Harding, Fujiwara, Axberg, & Harkonen, 2005). To do so, animals must balance their energy costs with the energy they extract from their environment (Kleiber, 1975). Bioenergetics integrates biotic and abiotic influences on the animals, external and internal factors, and can easily translate individual animal results into populations or community outcomes (Alunno‐Bruscia, van der Veer, & Kooijman, 2009). Energetic studies are now a primary tool used to investigate the effects of a wide range of environmental issues concerning the fitness of an individual and the dynamics of their populations (Grémillet, Wright, Lauder, Carss, & Wanless, 2003; Humphries, Umbanhowar, & McCann, 2004). However, at the core of these studies lies the fundamental question of how much energy animals require to freely live and thrive.

Metabolic rate and energy expenditure can be measured via respirometry (Withers, 1977), or more indirectly via heart rate frequency (Boyd, Bevan, Woakes, & Butler, 1999; Ceesey et al., 1989), or the differential elimination rates of H and O stable isotopes (doubly‐labelled water method, or DLW, Speakman, 1997). Respirometry is the most direct and accurate means to estimate gas exchange but is impossible to implement in most field studies. Although not as direct or accurate, heart rate and isotopic methods are more adapted to studies in wild settings and are often the only options available. However, they are not devoid of technical challenges and can be prohibitively costly (Butler, Green, Boyd, & Speakman, 2004). In addition, most of these methods only provide a gross estimate of energy expenditure over the time span of the measurement (days/weeks) when applied in the field (Butler et al., 2004).

Free‐ranging animals engage in numerous types of activities during their daily routine that are likely associated with different energetic costs (running, sleeping, eating, grooming, etc.). Consequently, understanding the behaviors and energetic needs of animals at a much finer scale would improve understanding of their physiology, biology, and ecology. New technologies, such as small biologging devices capable of recording triaxial acceleration and magnetic field at an unprecedented rate have opened the door to fine‐scale estimation of movements and positioning of animals in three dimensions (Nathan et al., 2012; Wilson et al., 2007). Along with recording geographical locations, depth and altitude, and other more traditional information, it is now possible to derive time–activity budgets, or proportions of time animals spend engaged in specific types of activity, at an unprecedented fine scale (Nathan et al., 2012).

It is challenging to estimate how much energy animals allocate to different activities and movements in the wild with the same degree of precision attained in captive studies. Metabolic costs associated with specific behaviors, or activity‐specific metabolic rates, have long been measured in captive or semi‐captive settings (Green, Halsey, Wilson, & Frappell, 2009; Jeanniard du Dot, Rosen, & Trites, 2009), but their transfer and applicability to wild animals are debated. With the emergence of newer technologies, methods of determining activity‐specific metabolic rates based on the link between dynamic body acceleration due to muscle work and energy consumption have been developed in terrestrial and marine species (Gleiss, Gruber, & Wilson, 2009; Wilson et al., 2006). However, most of these studies were performed in controlled or semi‐controlled settings, and their accuracy varies widely by species, gait, and environment (Elliott, Le Vaillant, Kato, Speakman, & Ropert‐Coudert, 2013; Halsey et al., 2008; Wright, Metcalfe, Hetherington, & Wilson, 2014). There is, thus, a need to develop and test methods to estimate energy costs at the activity level in wild settings that could be applied to different species.

**Figure 1:**
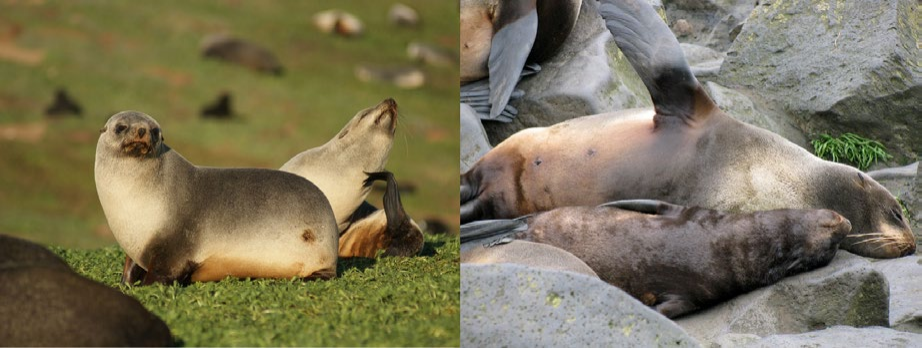
Antarctic fur seal female on Pointe Suzanne colony, Kerguelen Island, Southern Ocean (left), and northern fur seal female with pup on Reef rookery, St. Paul Island, Bering Sea (right) during their respective breeding season

The objectives of this study were to develop a method to estimate activity‐specific metabolic rates in wild free‐ranging animals in connection with their detailed time–activity budgets. We used northern and Antarctic fur seals—two sub‐polar species with similar physiology, biology, behavioral ecology, and reproductive strategy that have evolved in environments with similar features (Gentry & Kooyman, 1986) as biological models (Figure 1). The first step of our method requires implementing a decision tree algorithm to partition the time spent by individuals in different at‐sea activities (sleeping, diving, transiting, and resting) using fine‐scale data collected by biologging devices. The second step derives activity‐specific metabolic rates based on precise time–activity budgets and total at‐sea metabolic rates measured with a traditional and established method (DLW). We aimed to develop an energetic framework to estimate activity‐specific metabolic rates that can be applied to other marine and terrestrial species in hopes of furthering understanding of the interconnections between behaviors, energetics, and fitness—as well as providing a tool that can be used for conservation purposes to assess the consequences of environmental changes on foraging ecology, and help determine the underlying causes of population changes.

## 2 Material and Methods

### 2.1 Data collection

A sample from 20 lactating northern fur seals (NFS) was collected at Reef rookery on St. Paul Island (Bering Sea, 57°6′N–170°17′W) during the breeding season from Aug–Sep 2011, and from 20 lactating Antarctic fur seal (AFS) at Pointe Suzanne, Kerguelen Island (Southern Ocean, 49°26′S–70°26′E) during the breeding season from Jan–Feb 2012. Only females confirmed to be suckling a pup were selected for the study. Individuals were captured using a hoop net and were brought to a restraint board where they were anaesthetized with isoflurane gas. Standard morphometric measurements of length and axillary girth were made to the nearest 0.5 cm, and mass was recorded using a spring scale at ±0.2 kg. Data loggers were glued to the dorsal midline fur using a two‐part 5‐min epoxy glue. Daily Diary tags (DD, Wildlife Computers, Redmond, USA) recording triaxial acceleration at 16 Hz, and depth, light level, and water temperature at 1 Hz were glued between the scapulae of the animals. Fastloc^®^ GPS MK10 loggers (Wildlife Computers, Redmond, USA) were glued lower down the back from the DD tags and recorded GPS coordinates along the at‐sea track of the animal, as well as depth and water temperature at 1 Hz. Individuals were recaptured after a single foraging trip and anaesthetized as previously described to remove the data loggers and obtain biological samples.

### 2.2 Daily energy expenditure—doubly‐labelled water method

Measurements of daily energy expenditure (DEE, kJ/day) were performed using the DLW method (Butler et al., 2004; Lifson & McClintock, 1966). While animals were under anesthesia, an initial blood sample was taken by venipuncture on the hind flippers to determine ^2^H and ^18^O background levels. A weighed dose of DLW (0.3–0.6 g/kg body mass) was then intravenously injected via catheter into the other hind‐flipper before a second blood sample was taken after a 2‐hr period of equilibration. A final blood sample was taken to determine isotope levels of ^2^H and ^18^O at the end of the foraging trip upon recapture. We used a two‐pool model and a plateau method to calculate initial dilution spaces and the equation from Speakman, Nair, and Goran (1993) to account for evaporative water loss when calculating metabolic rates from DLW concentrations. Finally, we converted CO_2_ production rates into DEE using a respiratory quotient RQ of 0.80 (Dalton, Rosen, & Trites, 2014; Sparling, Thompson, Fedak, Gallon, & Speakman, 2008). See Supporting Information for details on DLW procedures and isotopic analyses.

The study individuals spent time on land after the postequilibration sample and upon return to the colony before they were recaptured, and the final blood samples were collected. Energy spent during this “non‐foraging” time was part of the DLW measurement. Thus, we calculated energy expenditure at sea by subtracting on‐land expenditure from the total estimate using previously determined values for lactating females in northern (4.67 W/kg in Gentry & Kooyman, 1986) and Antarctic fur seals (4.56 W/kg in Costa & Trillmich, 1988) while on land.

### 2.3 Diving and foraging behavior parameters

We used depth data recorded by the DD tags to determine diving behaviors, or depth data recorded by the Fastloc^®^‐MK10 when the DD tags malfunctioned. Any drift in the pressure sensors or error spikes were corrected prior to analyses. Diving behaviors were reconstructed using a custom‐made R program previously developed for Antarctic fur seals. Dives were defined as periods of time that animals spent underwater below a minimum depth of 3 m and for a minimum of 4 s until they returned to the surface. We derived dive duration and maximum dive depth for each of them.

### 2.4 Time–activity budget

**Figure 2:**
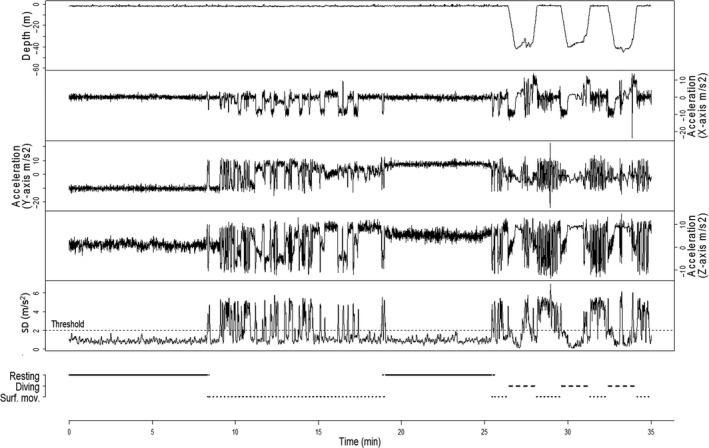
Example of depth profile, acceleration signals over the three axes for 35 min for a female northern fur seal foraging in the Bering Sea. Variance was calculated over the three acceleration channels, variance thresholds showing the level below which the animal was considered to be sleeping or resting (when the signal stayed below the threshold for more than 5 min at a time)

Fur seal behaviors were separated into four categories to determine time–activity budgets: (1) diving; (2) resting and sleeping; (3) surface activities, grooming, slow travel; and (4) fast transiting. These four behaviors were identified using a custom‐made classification tree algorithm in R, parameterized as follows:

1. *Diving* and foraging time were defined as the period when animals were actively diving (see *Diving and foraging behavior* parameters above) and included the postdive intervals as this is when the greatest elevation in MR occurs due to diving. Postdive intervals were defined as the interval between two successive dives during which time the animal returned to the surface to replenish its oxygen stores. Postdive intervals were estimated using the bout‐ending criterion (BEC) calculated with the maximum likelihood estimation method using the package diveMove in R (Author, S. Luque), validated for diving fur seals (Luque & Guinet, 2007).
2. *Resting* time was calculated by first applying a running standard deviation over 3 s on the raw acceleration of each of the three axes. The resulting signals were then averaged to obtain a single vector of same length as the initial ones. It was then defined as the time when the resulting average acceleration *SD* signal was <2.0 m/s^2^ for all three axes for >5 min (Figure 2). This acceleration threshold was determined visually and was similar for all animals (range of acceleration *SD* signals ~0.3–10 m/s^2^). Very short spikes in variance (of maximum 5 s) in the middle of long periods of variances (30 min or more) below the threshold of 2.0 m/s^2^ were considered to be due to the animal changing position while laying at surface and body orientation in the water (verified with the change in static acceleration) and were still considered in the resting time.
3. *Transiting* time was the period during which the animals were neither diving nor resting and were moving at the surface at ≥1 m/s. Surface speed was calculated by determining the distance between two successive GPS points and dividing it by the interval of time between them.
4. *Surface activities*, grooming and slow travel time occurred when the animals were neither diving nor resting and were moving at the surface at a speed <1 m/s.

Gaps in acceleration due to DD tags malfunction for northern fur seals were also quantified, and accuracy of the classification tree model was visually verified over the entire foraging trip for all animals.

### 2.5 Activity‐specific metabolic rates

Energy expenditure per type of activity was calculated by fitting the following model with general linear models (lm, “stats” package, R 3.0.3):

(1) $${\mathrm{EE}}_{\mathrm{Total}}=a\times T_{\mathrm{Dive}}+b\times T_{\mathrm{Transit}}+c\times T_{\mathrm{Surf}.}+d\times T_{\mathrm{Rest}}+e\times T_{\mathrm{Land}}$$

where EE_Total_ is the total energy expenditure in MJ measured from the DLW method for each seal, and *T*_*i*_, is the time in d it spent per activity (*i*), that is, diving (Dive), transiting (Transit), performing slow surface activities (Surf.), resting (Rest) and time spent on land (Land) before and after the foraging trip (included into the DLW method). This means that the parameter estimates *a, b, c, d,* and *e* for each type of activity are in MJ/day and correspond to the rate of energy expenditure for diving, transiting, surface activity, and resting, respectively. We consequently estimated the activity‐specific metabolic rates by fitting Equation (1) for all our seals to obtain the parameter estimates *a, b, c, d,* and *e*. If EE was estimated only for time at sea, the term [*e *× *T*_land_] was removed from Equation (1):

(2) $${\mathrm{EE}}_{\mathrm{At}\;\mathrm{Sea}}=a\times T_{\mathrm{Dive}}+b\times T_{\mathrm{Transit}}+c\times T_{\mathrm{Surf}.}+d\times T_{\mathrm{Rest}}$$

We forced the intercept through 0 because no energy is spent if no time is spent in any of the four defined activities. As R^2^ values are overinflated in models without intercepts, we computed the R^2^ as 1 − (RSS/ESS) as an additional means of comparison of the variance between models—where RSS is the regression sum of squares and ESS the error sum of square of the models. Normality, homogeneity of variances, and correlation between different explanatory variables were verified to ensure accordance with the model assumptions, even though the parameters *T*_*i*_ are not theoretically independent from one another. The models (Equations (1) and (2)) were compared to their respective NULL models which considered only “Total time” or “Time at sea” as the independent variable (still with no intercept). In models with more than two parameters, the sample size per species became too small and the power too low to obtain relevant statistics, so we pooled both species together. If one parameter estimate was not significant in the model output, it was removed from the equation and the simpler model was re‐run.

Statistical differences between two groups (e.g., between species, or between two activity types) were tested with two‐sample *t*‐tests (α = 0.05) or Mann–Whitney tests depending on normality.

## 3 Results

Eight DD tags failed totally or partially and seven females also returned to land with blood H and O isotopic levels that were too close to initial background levels to yield accurate metabolic rate measurements. They were removed from further analyses. Consequently, sample size for analyses was *n* = 12 for NFS and *n* = 13 for AFS.

Northern fur seal females weighed on average 37.9 ± 1.3 kg prior to departure and AFS females 31.0 ± 0.8 kg. There were no significant inter‐species differences for foraging trip duration 7.80 ± 3.11 days (range 2.34–15.47 days) and distance traveled while foraging 692 ± 292 km (range 225–1,295 km, both *p *> .221). Rates of energy expenditure during the entire foraging trip at sea (i.e., including a range of different activities) were also not statistically different between species whether for the at‐sea time only (at‐sea field metabolic rate =19.28 ± 5.49 MJ/day) or when time on land was included (18.47 ± 4.94 MJ/day, both .08 < *p *< .06). Finally, both species spent the same amount of energy during their foraging trip at sea (137.75 ± 63.53 MJ, *p *>* *.09).

### 3.1 Time–activity budget

Northern and Antarctic fur seals allocated similar time to diving (28.84 ± 6.15%, range 20.57–47.81%, *p* = .328), transiting to and from their foraging grounds (28.93 ± 7.94%, range 15.27–46.61% *p* = .063), and resting and sleeping (9.06 ± 6.55%, range 1.25–24.60%, *p *= .248). However, Antarctic fur seals spent more time grooming and moving slowly at the surface than northern fur seals (28.8 ± 1.4% for NFS and 36.3 ± 2.0% for AFS, range 15.97–47.66%, *p* = .013). Foraging trip duration and proportion of time spent engaged in different activities did not depend on mass of the animals (all *p *> .3). As foraging trip duration varied between individuals (range 2.34–11.86 days), the absolute time animals spent diving ranged from 0.62 to 3.24 days, transiting from 0.70 to 4.84 days, performing slow surface movements from 0.82 to 4.65 days, and resting from 0.01 to 1.02 days (see Table S1 in Supporting Information).

### 3.2 Activity‐specific metabolic rates

Models from Equations (1) and (2) were fit with measures of energy expenditure for each seal and with their respective time at sea (NULL model) or with their time–activity budgets. The two species were pooled because statistical power was too low for the separate groups. As explanatory variables are in days at sea and the response variable is in MJ, parameter estimates for each variable indicate the rate of energy expenditure in MJ/day for each type of activity (Table 1). Models involving time–activity budgets were better predictors of energy expenditure than model with only total time at sea or total time of DLW measurement (Table 1).

**Table 1:** Parameters for general linear models describing the relationship between energy expended by northern and Antarctic fur seals per day to complete a single foraging trip (in MJ) as well as the cost of different foraging activities (time spent foraging, transiting, performing surface movements/grooming, or resting). Note that “Total time” includes time at sea and time on shore tending to pups (i.e., one foraging cycle), while “Time at sea” only accounts for the time away from land. These models were also fit with (upper rows) or without (lower rows) resting time

Dependant variable	Parameter (days)	Estimate (MJ/day)	SE	*p*	R^2^	AICc
EE total time (MJ)	Total time	18.58	0.88	<10^−15^	0.65	265.4
EE total time (MJ)	Diving	29.96	6.02	<10^−4^	0.64	268.1
	Transiting	21.55	7.49	0.004		
	Surf. Mov.	13.85	8.24	0.105		
	Land	13.11	7.02	0.068		
	Resting	−2.34	32.69	0.942		
EE at sea (MJ)	Time at sea	19.47	1.05	<10^−15^	0.64	264.6
EE at sea (MJ)	Diving	30.92	5.77	<2.10^−5^	0.70	265.7
	Transiting	18.66	6.80	0.012		
	Surf. Mov.	14.95	7.93	0.073		
	Resting	−3.04	30.67	0.922		
EE total time (MJ)	Diving	29.93	5.87	<10^−4^	0.70	266.1
	Transiting	21.49	7.27	0.007		
	Surf. Mov.	13.49	6.36	0.045		
	Land	12.98	6.63	0.063		
EE at sea (MJ)	Diving	30.84	5.62	<10^−4^	0.70	263.7
	Transiting	18.50	6.48	0.009		
	Surf. Mov.	14.47	6.18	0.028		

The two major drivers of energy expenditure in fur seals were diving and transiting (Table 1). Outputs from Equation (1) provided a negative parameter estimate for resting activity (Table 1). However, standard errors for the term were more than 10‐fold greater than the estimate itself, and values did not differ significantly from 0. In addition, the *p* values associated with this activity were >0.05. We consequently excluded resting and sleeping time from Equation (1). The simpler model did not fit the data better than the full model (Equation (1); *F*‐test *p > *.922), but had a lower AIC value (AIC = 263.7, Table 1). It was thus considered the most parsimonious one. Consequently, it was possible to calculate energy spent while performing each activity type (in MJ) by multiplying the parameter estimates from Equation (1) (in MJ/day, see Table 1) by individual seal time–activity budgets (in days).

The relationship between time–activity budget and energy spent during the period between the two DLW measurements (i.e., including energy spent during the time females were on land before and after their foraging trip in between capture and recapture) showed similar trends and parameter estimates compared to time at‐sea alone. Rate of energy expenditure for time resting on land equaled the rate of energy spent at‐sea engaged in grooming and slow surface movements (13–14 MJ/day, Table 1).

## 4 Discussion

Time and energy define bioenergetics and determine the likelihood of individuals surviving and successfully reproducing. However, it is notoriously difficult to measure these highly intertwined and closely cross‐regulated currencies in the daily lives of animals. In this study, we combined fine‐scale behavioral data and total energy spent to derive metabolic rates associated with different at‐sea activities of animals. To do so, we analyzed the fine‐scale time–activity budgets of free‐ranging female northern and Antarctic fur seals (i.e., how much time they spent engaged in traveling, diving, grooming, and resting) and used these to calculate metabolic rates for each of these activities given the total energy expended. Our study showed that activity‐specific metabolic rates can be calculated accurately for wild animals which opens the door to a more fine‐scale understanding of how individuals control their energy and time balances.

Our study focused on two closely related species of fur seals, the northern and the Antarctic fur seals. Our statistical power was too low to fit Equation (1) separately by species given the failures in some of the biologgers and the DLW measurements. However, both species were consistently similar in both energetics and behaviors measured throughout the course of this study (discussed below), which supports our choice of pooling them for analyses and, thus, increasing our power for statistical analyses. Indeed, the average daily metabolic rate (MR_sea,_ measured in situ from the DLW method) during foraging trips was similar for both species at 4.7 ± 0.3 × BMR (basal metabolic rate in kJ/day calculated from Kleiber's equation used for easier comparisons, Kleiber, 1975). This is consistent with previously reported metabolic rates for Antarctic fur seals (4.59 ± 0.32 × BMR from Arnould, Boyd, & Speakman, 1996) and California sea lions *Zalophus californianus* (5.2 × BMR from Boyd, Woakes, Butler, Davis, & Williams, 1995; 4.8 × BMR from Ponganis, Kooyman, Winter, & Starke, 1997), although lower than the earlier 6.16 × BMR for northern fur seals (Costa & Gentry, 1986) and 6.7 × BMR for Antarctic fur seals (Costa, Croxall, & Duck, 1989) that were later considered to be overestimates (Butler, Bevan, Woakes, Croxall, & Boyd, 1995).

Similarly, duration of foraging trips at sea were approximately 7.5–8.0 days for both species. More surprisingly, both species of fur seals also showed very similar time–activity budgets despite living in habitats at opposite latitudes (although with very similar environmental features). They spent ~29% of their time diving, 25%–30% transiting, 29%–36% involved in surface activities, and 8%–10% sleeping. This is again consistent with earlier studies. For example, female northern fur seals were previously found spending 23%–35% of their time diving (Costa & Gentry, 1986; Insley, 2008) and Antarctic fur seals ~19% (Bailleul, Luque, Dubroca, Arnould, & Guinet, 2005). These authors also reported 44%–60% of time swimming and moving, and 5%–33% of time resting for northern fur seals and 54% swimming, and 23% resting for Antarctic fur seals. These numbers can seem to deviate from our estimates, but it is important to keep in mind that our time–activity budget is more detailed as we added the “surface activity” category to our list which splits the time not spent diving into three categories instead of two for these studies.

Once fine‐scale time–activity budgets were determined, we calculated metabolic rates specific to each type of activity from a best fit model using total energy expenditure at sea measured from the DLW method (Equation (1)). The metabolic rates we calculated for each type of activity were well in accordance with previously reported measured values. First, our calculated metabolic rate on land was ~3.3 ± 0.1 × BMR, which is close to the 3.2 × BMR metabolic rate of female Antarctic fur seals and to the 3.4 × BMR metabolic rate of female northern fur seals nursing on land (Costa & Trillmich, 1988; Gentry & Kooyman, 1986). As metabolic rate on land is the only “activity‐specific” metabolic rate from our model that was previously measured in the wild independently of other activities, the close concordance in calculated versus previously measured values provides confidence in the results of our model. Second, we calculated that the most expensive activity the fur seals engaged in was diving, which was ~7.5 × BMR. This is close to underwater swimming metabolic rate in southern sea lions and California sea lions (6.8 × BMR from Boyd et al., 1995; Dassis, Rodríguez, Ieno, & Davis, 2012). Transiting at the surface (surface movement ≥1 m/s) was 4.5 ± 0.4 × BMR, which was less expensive than diving and equivalent to our average daily metabolic rate at sea (4.5 ± 0.2 × BMR for all seals). Butler et al. (1995) also found that metabolic rate while diving was greater than when transiting by an estimated 20% in Antarctic fur seals. In our case, diving was nearly 30% more costly than transiting at the surface. Transiting fur seals usually porpoise at the surface or swim at depths equivalent to three times their body diameters, which would reduce drag forces and cost of transportation compared to diving (Boyd, 2002; Hindle, Rosen, & Trites, 2010).

Arnould et al. (1996) found that energy expenditure at sea was negatively related to proportion of time spent diving in Antarctic fur seals and, thus, concluded that the costliest activity was transiting rather than diving. We did not observe this relationship in our data. They, however, had a smaller sample size for their analyses, and only estimated time spent diving (using depth recorded every 10 s). They also did not separate the rest of their time at sea into transiting or resting. The finer scale and more detailed time–activity budget in our study might explain the difference in the results and highlights the added value of fine‐scale behavioral data provided by accelerometers to refine these budgets.

Overall, the similarities between our field and modeled estimates of metabolism indicate that our models provide accurate activity‐specific metabolic rates. It would have been mathematically equivalent to fit Equation (1) with mass‐specific energy expenditures, but this would imply that future users of the models would have knowledge of their animal's mass, which is not always the case in field studies. In addition, neither species nor the mass and size of females affected foraging trip duration, behaviors at sea or time partitioning between different types of activities. As a consequence, mass was not significant in our calculations of activity‐specific metabolic rates from Equation (1). We, thus, obtained results for the mass range of our study animals.

One interesting point is that the calculated metabolic rate associated with time spent resting was neither accurate nor significant in our model. The standard error associated with the sleeping metabolic rate parameter estimates are one order of magnitude greater than the estimate itself. This does not mean that sleeping metabolic rate is biologically non‐existent at sea. However, within the context of the full foraging trip and the model tested (and with our statistical power), the importance of the metabolic rate of sleeping is not significant. This is likely due to a combination of sleeping being the least expensive activity at sea and the fact that animals spent the least amount of time engaged in this activity at sea on average despite this parameter being the most variable between individuals.

Energy expenditure can vary within a specific activity and over the timescale of foraging trip for a given activity (Costa, 2008). Unfortunately, the data collected and our sample size did not allow metabolic rates to be estimated at this level of detail. Individual differences in foraging strategies can also influence energetics of animals (Boyd, 1999; Costa & Gentry, 1986). In our study, northern fur seals foraged in the relatively shallow waters of the Bering Sea continental shelf (*n* = 9 in the present study) or in the deep waters off the shelf (*n* = 4), but had similar field metabolic rates over duration of their foraging trips (NFS 0.56 ± 0.05 vs. AFS 0.54 ± 0.07 MJ/day/kg). We thus pooled all animals together for our analyses irrespective of their foraging strategy. Our study intended to provide average activity‐specific metabolic rates for the population given the variations in time–activity budgets, foraging strategies and trip durations between individuals. As such, our estimates encompass these different levels of variations and provide generic estimates for average northern and Antarctic fur seals. Furthermore, yearly variations in metabolic rates depending on environmental conditions and prey availability (Boyd, 1999) usually result from a change in time–activity budget, that is, an increase in the time looking for prey before an increase in duration of their trips (Arnould et al., 1996; Costa, 2008) during years of difficult conditions. Even though our study only spanned one year, impacts of changes in environmental conditions on energy balance of animals should thus also be at least partially estimated using our average activity‐specific metabolic rates by quantifying these time–activity budgets and how they vary.

To conclude, our study presents a new method to determine metabolic rates at the activity level for wild animals engaged in different activities within their daily lives. It shows how the allocation of time and energy are regulated in parallel and how intimate knowledge of one leads to a greater understanding of the other. Our framework could provide information needed to understand the mechanisms by which individuals and populations adjust, or fail to adjust, to environmental changes given that environmental changes affect time–activity budgets and energetic balance (Arnould et al., 1996; Costa, 2008) that in turn affect fitness (Lescroël et al., 2010; Jeanniard du Dot, 2015). Our framework could also be useful for predictive individual‐based bioenergetic models, or for assessing energy flow within ecosystem bioenergetic models by giving insight into the energy needs of individuals that have to be balanced with energy intakes.

The scientific interest in determining activity‐specific energetic costs of free‐ranging animals has been long standing. The relatively simple method we propose to calculate activity‐specific metabolic rates based on detailed activity budgets and global field metabolic rates of individuals has the potential to yield better and more fine‐scale understanding of the lives of wild animals—whether terrestrial or marine—and the consequences that changes in behaviors have on their physiologies and abilities to meet their energetic needs.

## Conflict of Interest

None declared.
