# Supplementary material for: Activity‐specific metabolic rates for diving, transiting, and resting at sea can be estimated from time–activity budgets in free‐ranging marine mammals
Source: Ecol Evol. 2017 Mar 23;7(9):2969–76. doi: 10.1002/ece3.2546 (PMC5415512; doi:10.1002/ece3.2546)
Supplement: Supplementary file 1 [file ECE3-7-2969-s001.docx]

# Supporting information

Details on the DLW field and laboratory procedures:

Measurements of daily energy expenditure (DEE, kJ/day) were performed using the Doubly-Labeled Water (DLW) method ([Lifson & McClintock 1966](#_ENREF_30); [Butler](#_ENREF_9) *[et al.](#_ENREF_9)* [2004](#_ENREF_9)). We took a first blood sample (3 to 8ml) by veinipuncture of an inter-digital vein in a hind-flipper to determine baseline levels of ^2^H and ^18^O for each individual ([Speakman & Racey 1987 – method D](#_ENREF_43)). A known mass pulse-dose of DLW (622272 ppm ^18^O, 384645 ppm ^2^H) was then administered intravenously via a catheter on the other hind-flipper (0.3-0.6g/kg body mass) and flushed with saline solution to ensure full injection into the blood stream. Syringes were weighed before and after administration (±0.0001g, Sartorius balance) to calculate the mass of DLW injected. The labelled isotopes were allowed to equilibrate with the body water pool for 2h during which the seals were either kept under very light anaesthesia or kept in a quiet closed environment. Equilibration times have previously been determined by serial blood sampling to be less than 2 hours on fur seals ([Costa 1987](#_ENREF_11); [Arnould 1995](#_ENREF_2)). Equilibration times have previously been determined by serial blood sampling to be less than 2 hours on fur seals ([Costa 1987](#_ENREF_11); [Arnould 1995](#_ENREF_2)). At the end of this period, a second blood sample was taken.

All blood samples were collected in Monovette syringes (Sarstedt) coated with Li-Heparin and containing a plasma-red blood cells separator. Plasma was isolated from red blood cells either by centrifugation at 1000g for 10 min (northern fur seals), or by natural gravity separation for 4 hours when no electricity was available to power a centrifuge in the field (Antarctic fur seals). Plasma samples were then flame sealed into 2 x 100µL glass capillary tubes, and stored at room temperature until isotopic analyses were performed. For isotopic analyses, plasma samples were vacuum distilled ([Nagy 1983](#_ENREF_32)) and the resulting distillate was used to produce CO_2_ and H_2_ ([methods in Speakman 1990 for CO2](#_ENREF_38) ; [and Speakman & Krol 2005 for H2](#_ENREF_41)). The isotope ratios ^18^O: ^16^O and ^2^H: ^1^H were analysed using gas source isotope ratio mass spectrometry (Optima, Micromass IRMS and Isochrom μG, Manchester, UK). Samples were run alongside three lab standards for each isotope (calibrated to International standards) to correct delta values to ppm. Isotope enrichments were converted to CO_2_ production for each individual using a two-pool model (i.e. considering respective individual dilution spaces for ^18^O and ^2^H), best suited for larger animals including pinnipeds ([Speakman 1987](#_ENREF_37); [Schoeller 1988](#_ENREF_35); [Sparling *et al.* 2008](#_ENREF_36)). Initial isotope dilution spaces were calculated using the plateau method ([Halliday & Miller 1977](#_ENREF_22)).

Finally, several approaches can be taken to account for evaporative water loss when calculating metabolic rates from DLW concentrations ([Visser & Schekkerman 1999](#_ENREF_44)). We used 4 equations for our calculations: the equation 7.43 in Speakman ([1997](#_ENREF_40)) that assumed evaporation of 25% of the water flux which minimizes error in a range of conditions and were deemed most appropriate in a validation study on seals ([Sparling *et al.* 2008](#_ENREF_36)); and the equations from Speakman ([1993](#_ENREF_39)), [Speakman, Nair and Goran (1993](#_ENREF_42)) and [Coward *et al.* (1985](#_ENREF_15)) that were recently shown to be the most accurate on captive northern fur seals in summer and fall when compared to metabolic rate measurements via respirometry ([Dalton, Rosen & Trites 2014](#_ENREF_16)). We however only report the best results from [Speakman, Nair and Goran (1993](#_ENREF_42)) as assessed in [Jeanniard du Dot (2015](#_ENREF_27)). Finally, we converted CO_2_ production rates into daily energy expenditure using a respiratory quotient (RQ = O_2_ consumption / CO_2_ production) of 0.80 as an estimate based on measurements performed in spring and summer (0.80) and fall (0.77) on northern fur seals ([Dalton, Rosen & Trites 2014](#_ENREF_16)) and on grey seals on a typical fish diet ([0.76, Sparling *et al.* 2008](#_ENREF_36)).

Table S1: Metabolic and behavioural parameters for 12 northern fur seal and 13 Antarctic fur seal females collected during 1 foraging trip at sea during the breeding season 2011-2012 on St Paul Island, Bering sea, and Kerguelen Island, Southern Ocean.

| **Species** | **Trip duration (d)** | **Mass Change (kg)** | **Total energy expenditure (MJ)** | **Diving (d)** | **Transiting (d)** | **Surface movement (d)** | **Resting (d)** |
| --- | --- | --- | --- | --- | --- | --- | --- |
| NFS | 8.39 | 9.9 | 285.46 | 2.39 | 2.34 | 2.54 | 1.03 |
| NFS | 9.36 | 1.7 | 184.28 | 2.26 | 3.02 | 3.00 | 1.00 |
| NFS | 6.84 | 10.5 | 207.44 | 1.94 | 1.50 | 2.50 | 0.89 |
| NFS | 5.50 | -9.9 | 155.70 | 1.27 | 2.14 | 1.54 | 0.54 |
| NFS | 4.36 | -5.9 | 124.75 | 2.08 | 0.92 | 0.85 | 0.48 |
| NFS | 7.30 | 11.8 | 211.29 | 1.67 | 2.50 | 2.51 | 0.48 |
| NFS | 7.97 | -4.7 | 192.20 | 3.18 | 2.46 | 1.79 | 0.44 |
| NFS | 8.35 | -0.6 | 145.76 | 1.97 | 2.85 | 2.41 | 0.94 |
| NFS | 11.86 | 15.5 | 261.98 | 2.99 | 4.84 | 2.54 | 0.12 |
| NFS | 9.40 | -2.5 | 201.58 | 1.93 | 4.38 | 2.57 | 0.48 |
| NFS | 5.85 | 3.3 | 176.34 | 2.51 | 1.65 | 1.16 | 0.43 |
| NFS | 7.80 | -4.9 | 166.66 | 1.68 | 2.42 | 2.72 | 0.97 |
| AFS | 8.91 | 12.3 | 238.51 | 2.68 | 2.97 | 2.67 | 0.59 |
| AFS | 6.35 | 0.0 | 133.27 | 1.67 | 2.18 | 2.27 | 0.24 |
| AFS | 3.30 | 0.0 | 79.17 | 0.97 | 0.93 | 1.21 | 0.19 |
| AFS | 6.16 | 9.4 | 79.83 | 1.86 | 0.97 | 2.93 | 0.39 |
| AFS | 3.49 | 0.0 | 66.76 | 0.85 | 0.91 | 1.22 | 0.51 |
| AFS | 2.34 | -6.5 | 57.03 | 0.63 | 0.74 | 0.96 | 0.01 |
| AFS | 3.04 | 0.0 | 69.32 | 1.00 | 0.70 | 0.83 | 0.52 |
| AFS | 3.90 | -2.6 | 78.95 | 1.15 | 1.08 | 1.49 | 0.19 |
| AFS | 4.20 | 5.6 | 115.92 | 1.28 | 0.86 | 1.88 | 0.18 |
| AFS | 4.93 | 11.5 | 93.68 | 1.29 | 1.04 | 2.25 | 0.36 |
| AFS | 8.85 | 8.3 | 134.90 | 3.05 | 2.89 | 2.20 | 0.71 |
| AFS | 8.09 | 0.0 | 211.26 | 2.65 | 1.24 | 3.75 | 0.46 |
| AFS | 11.10 | 2.9 | 214.02 | 3.24 | 2.28 | 4.65 | 0.93 |
